# The effect of physical activity on anxiety through sleep quality among Chinese high school students: evidence from cross-sectional study and longitudinal study

**DOI:** 10.1186/s12888-025-06909-x

**Published:** 2025-05-16

**Authors:** Xianghe Chen, Yuxin Yang, Chenghao Zhong, Xinyu Zeng, Xiao Qiu, Xiangxiang Zhou, Chi Liu, Zhikai Tian, Bo Liu, Rongbin Yin

**Affiliations:** 1 College of Physical Education, Yangzhou University, Yangzhou, 225127 China; 2 JiangSu College of Tourism, Yangzhou, 225000 China; 3 Physical Education and Sports School of Soochow University, Suzhou, 215021 China

**Keywords:** Physical activity, Sleep quality, High school students, Anxiety

## Abstract

**Background:**

Anxiety disorder is a significant concern in the context of mental health among Chinese high school students. Based on cross-sectional study and longitudinal study, this study constructed mediation models in order to evaluate the mediation effects of sleep quality in the improvement of anxiety by physical activity among Chinese high school students.

**Methods:**

A total of 32,974 Chinese high school students were surveyed using the International Physical Activity Questionnaire (IPAQ), the Pittsburgh Sleep Quality Index (PSQI), and the Generalized Anxiety Disorder 7-item Scale (GAD-7). We conducted the survey to construct a mediation model of anxiety, and then verified it with longitudinal data. After 12 weeks of exercise, 80 high school students were tested for physical activity level, sleep quality score and anxiety level.

**Results:**

The mediation model based on cross-sectional study showed a good fit with the data. Physical activity significantly positively predicted sleep quality but negatively predicted anxiety. Sleep quality significantly negatively predicted anxiety and had a mediation effect between physical activity and anxiety. Further longitudinal study proved that physical activity could improve both sleep quality and anxiety among high school students.

**Conclusions:**

Evidence from cross-sectional study and longitudinal study indicated the mediation role of sleep quality in the relationship between physical activity and anxiety among high school students, providing a theoretical and practical basis for physical exercise to improve high school students’ anxiety and other psychological problems. Besides, physical activity could relieve anxiety through sleep quality, but causality cannot be inferred when using only a cross-sectional study design.

**Supplementary Information:**

The online version contains supplementary material available at 10.1186/s12888-025-06909-x.

## Introduction

Chinese high school students are exposed to a confluence of psychosocial stressors spanning societal expectations, academic pressures, familial dynamics, and personal developmental challenges, contributing to elevated risks of anxiety disorders and related psychological comorbidities [1]. The ramifications of adolescent anxiety extend beyond impaired academic performance to encompass severe mental health sequelae, including major depressive episodes and suicidality, thereby emerging as a critical public health priority. In alignment with national strategic frameworks—specifically the Healthy China Initiative (2019–2022) and the Outline of the Healthy China 2030 Plan—there is an urgent imperative to implement comprehensive school-based mental health interventions. These initiatives explicitly emphasize the integration of evidence-based preventive strategies targeting anxiety mitigation within secondary education systems. Based on this, a growing body of evidence has revealed that achievement motivation, emotional regulation, academic pressure and social support are closely related to anxiety in high school students, and sleep quality is a classic predictor of anxiety. The Eighth National Student Physical Health Survey Report documents a concerning prevalence of sleep disorders among Chinese high school students, a finding corroborated by independent meta-analytic evidence reporting an incidence rate of 24.1% [2]. Notably, 35.7% of this population exhibits persistent sleep disturbances [3], with epidemiological patterns demonstrating significant rural-urban disparities [4]. Furthermore, the observed mean sleep duration of 7.41 h systematically deviates from the nationally recommended 8-hour standard [5]. Currently, researchers have restricted most of the studies on the relationship between sleep quality and anxiety to middle school and college students, and they have conducted few studies on high school students. Recent studies increasingly suggest that depression is not a static psychological state but a dynamic process, with its manifestations and severity changing over time. While many cross-sectional studies focus on depressive states at a single point in time, longitudinal data have been used to reveal the dynamic characteristics of depression. These studies have identified significant heterogeneity in depressive trajectories across different populations, indicating that the development of depression is influenced by various factors, including individual differences, environmental stressors, and social support. For example, a 2023 study found that the relationship between physical activity time, openness, and depressive symptoms among Chinese adolescents varied over time [6]. Another study in 2024 revealed significant differences in mental health experiences among college students from diverse backgrounds [7]. Additionally, Musliner et al. (2016) identified multiple subtypes of depressive symptom trajectories, including chronic, recurrent, and remitting depression, by analyzing longitudinal data [8]. These findings underscore the high degree of individual variability in depression development and the potential for significant differences in depressive manifestations across populations. Similarly, Nandi et al. (2009) noted that socioeconomic status, gender, and age influence long-term depressive trajectories, further supporting the heterogeneity of depression [9]. Moreover, Kessler et al. (2010) demonstrated that the onset and remission of depression are dynamic processes, with significant variations in symptom patterns across subgroups [10]. These findings highlight that the heterogeneity of depression is evident not only in symptom severity but also in developmental trajectories and influencing factors. Given the unique circumstances of Chinese students, understanding the connections between physical activity, sleep quality, and anxiety in this population is crucial for improving mental health promotion strategies. The inverse correlation between sleep quality and anxiety is mediated through neurochemical pathways involving diminished 5-hydroxytryptamine (5-HT) signaling, which attenuates ventral tegmental area (VTA) and lateral hypothalamic area (LH) activation, coupled with γ-aminobutyric acid (GABA)-mediated suppression of glutamatergic transmission and subsequent neuronal apoptosis in cortical perfusion zones [11–13]. Conversely, anxiety states may potentiate the initiation and progression of sleep disorders through feedforward neuroendocrine mechanisms [14]. The present investigation specifically examines the unidirectional impact of sleep disturbances on anxiety development in adolescents, with the primary hypothesis (H1) postulating that diminished sleep quality negatively predicts anxiety severity.

As a modifiable behavioral intervention, structured physical exercise demonstrates significant predictive validity for sleep health parameters, accounting for 22.9% of the variance in subjective sleep quality, sleep maintenance efficiency, and daytime functioning among adolescents when implemented consistently during both academic and non-academic days [15]. Physical activity, distinguished from regimented exercise by its incorporation of spontaneous movement patterns, provides a more comprehensive metric for assessing adolescent energy expenditure profiles [16], thereby gaining increasing research attention. Empirical evidence reveals robust inverse associations between physical activity levels and key sleep pathology indicators across developmental stages, including reduced sleep onset latency, decreased sleep schedule variability, and lower global scores on standardized sleep assessments such as the Children’s Sleep Habits Questionnaire (CSHQ) [17]. This relationship extends beyond pediatric populations, with insufficient physical activity strongly associated with sleep architecture disturbances in adolescents [18], university cohorts [19], and geriatric populations [20]. Previous research has shown that physical exercise can reduce insomnia levels in adolescents by enhancing psychological flexibility and alleviating stress [21]. Physical exercise can also affect the sleep quality of adolescents by reducing anxiety and mobile phone dependence (and may even exert its influence entirely through these mediating variables) [22]. Both physical exercise and physical activity are able to enhance sleep quality, and physical exercise is the key means to improve the level of physical activity, but the relationship between physical activity and sleep quality of high school students remains to be understood. Therefore, our second hypothesis (H2) is physical activity positively predicting sleep quality of high school students.

According to the person-environment (P-E) fit and cognitive-behavioral model of pathological Internet use (PIU), physical activity is an important factor in relieving anxiety. The level of physical activity shows gender difference, and the level of physical activity of Grade 11 students is the highest and that of Grade 12 students is the lowest. The low level of health fitness caused by long-time sitting makes the level of anxiety significantly higher [23]. Several studies have confirmed that physical activity can significantly improve anxiety among college students [24, 25], while there are fewer studies on high school students. In terms of students in Grade 12, level of physical activity in general is moderate, 29% of them have low physical activity, 24% have moderate anxiety, and 12% have severe anxiety [26]. In previous studies on adolescents, some have considered the role of physical exercise in alleviating adolescent anxiety to achieve a reduction in the level of Internet addiction [27, 28]. However, no known empirical research has focused on exploring relationship between physical activity and the incidence of anxiety in high school students. Therefore, our third hypothesis (H3) is physical activity negatively predicting anxiety of high school students.

The stress-buffering theory of physical activity posits that as a critical psychophysiological resource, regular engagement enhances multisystem functioning and psychological resilience, thereby mitigating the development of negative emotional states [29]. Empirical evidence consistently demonstrates a significant inverse association between sleep quality and anxiety severity [30], with sleep parameters serving as pivotal mediators in physical activity-induced anxiety reduction. This mechanistic pathway is corroborated by meta-analytic evidence indicating moderate-effect anxiety alleviation in collegiate populations through sleep quality improvement [31]. Current research predominantly examines the physical activity-sleep-anxiety nexus within collegiate populations [26, 29]. Therefore, our fourth hypothesis (H4) is sleep quality has a mediating effect between physical activity and anxiety among high school students.

Guided by the theoretical integration of positive psychology and psychophysiology, this study establishes and validates a mediation model delineating the psychophysiological pathway through which physical activity—as a modifiable behavioral intervention—ameliorates adolescent anxiety via enhancement of sleep quality as a core protective resource.

The specific research hypotheses proposed in this paper include the following:

### H1

Sleep quality negatively predicts anxiety.

### H2

Physical exercise positively predicts sleep quality of high school students.

### H3

Physical activity negatively predicts anxiety in high school students.

### H4

Sleep quality plays a mediating role between physical activity and anxiety in high school students.

## Methods

### Participants

To address these issues and explore the underlying mechanisms, this study focuses on the relationship between physical activity, sleep quality, and anxiety among high school students. A stratified, multistage, cluster sampling method was used to select 129 high schools across 13 cities in Jiangsu Province, ensuring representation of diverse educational environments and socioeconomic backgrounds. Schools were stratified based on factors such as school level (e.g., key schools vs. ordinary schools) and urban-rural distinctions. We randomly chose a total of 129 high schools in 13 cities of Jiangsu Province and used stratified random sampling to select 300 males and 300 females from Grade 10 to Grade 12 (Aged 14–20 years old) in each school. A total of 40,000 paper-based questionnaires were distributed on-site, with 36,573 valid responses collected, yielding an effective recovery rate of 91.4%.

Inclusion criteria for participants were: (1) voluntary participation with written informed consent from both students and their guardians; (2) enrollment in ordinary high schools; (3) aged 14–20 years; and (4) no motor disorders or addictions (e.g., smoking or alcohol). Exclusion criteria included: (1) questionnaires completed in less than 180 s; (2) incomplete or inconsistent responses; and (3) obvious logical errors in responses. This study is an “independent register” which was approved by the Institutional Review Board of Yangzhou University (Approval No.YZUHL2021010).All participants and their guardians signed informed consent and could withdraw at any time during the study. All personal identifying information was stored separately from research data, with access restricted to authorized team members who signed confidentiality agreements. Data were anonymized using unique codes, ensuring that participants’ identities could not be traced during analysis. In reporting findings, no identifiable information was disclosed, adhering to academic and ethical standards. Participants were fully informed about the study’s purpose, procedures, and their rights, and written consent was obtained prior to participation.

Data collection was conducted using three validated scales: the International Physical Activity Questionnaire Scale (IPAQ) to assess physical activity levels, the Pittsburgh Sleep Quality Index Scale (PSQI) to evaluate sleep quality, and the Generalized Anxiety Disorder 7-item Scale (GAD-7) to measure anxiety symptoms. Basic demographic information, including gender, grade, residential area, and whether the participant was the only child in the family, was also collected. To ensure data integrity, a rigorous screening process was implemented: (1) questionnaires with a large number of unanswered questions were excluded; (2) short responses (completed in less than 180 s) were removed to avoid random responses; and (3) questionnaires with obvious logical errors were investigated and excluded. After screening, 32,974 valid questionnaires remained, with an effective recovery rate of 90.2%. The final sample included 16,896 males and 16,078 females, distributed across Grade 10 (10,974 students), Grade 11 (9,841 students), and Grade 12 (12,159 students).

The study incorporated a comprehensive set of covariates to control for potential confounding factors. These included: (1) Demographic variables: gender (male or female), grade (Grade 10, Grade 11, and Grade 12), whether the participant was the only child in the family (yes or no), and residential environment (urban or rural residence). (2) Physical activity levels: classified as low, moderate, or high based on IPAQ scores. (3) Sleep-related variables: sleep quality, time to fall asleep, sleep duration, sleep efficiency, sleep disorders, hypnotic drugs, and daytime dysfunction based on PSQI scores. (4) Anxiety levels: categorized as no anxiety symptoms, mild anxiety, moderate anxiety, or severe anxiety based on GAD-7 scores. These covariates were included to ensure a robust analysis of the relationships between physical activity, sleep quality, and anxiety, while accounting for potential confounding influences.

To further validate the mediation model, We conducted exercise interventions on 80 Grade 11 students (40 for each gender) of Tianjiabing Senior High School in Changzhou City. Inclusion criteria for the intervention group were: (1) aged 15–17 years; (2) healthy with no motor disorders; and (3) no history of smoking or alcohol addiction. The intervention aimed to explore the causal relationships between physical activity, sleep quality, and anxiety, providing additional insights into the underlying mechanisms.

**Fig. 1:**
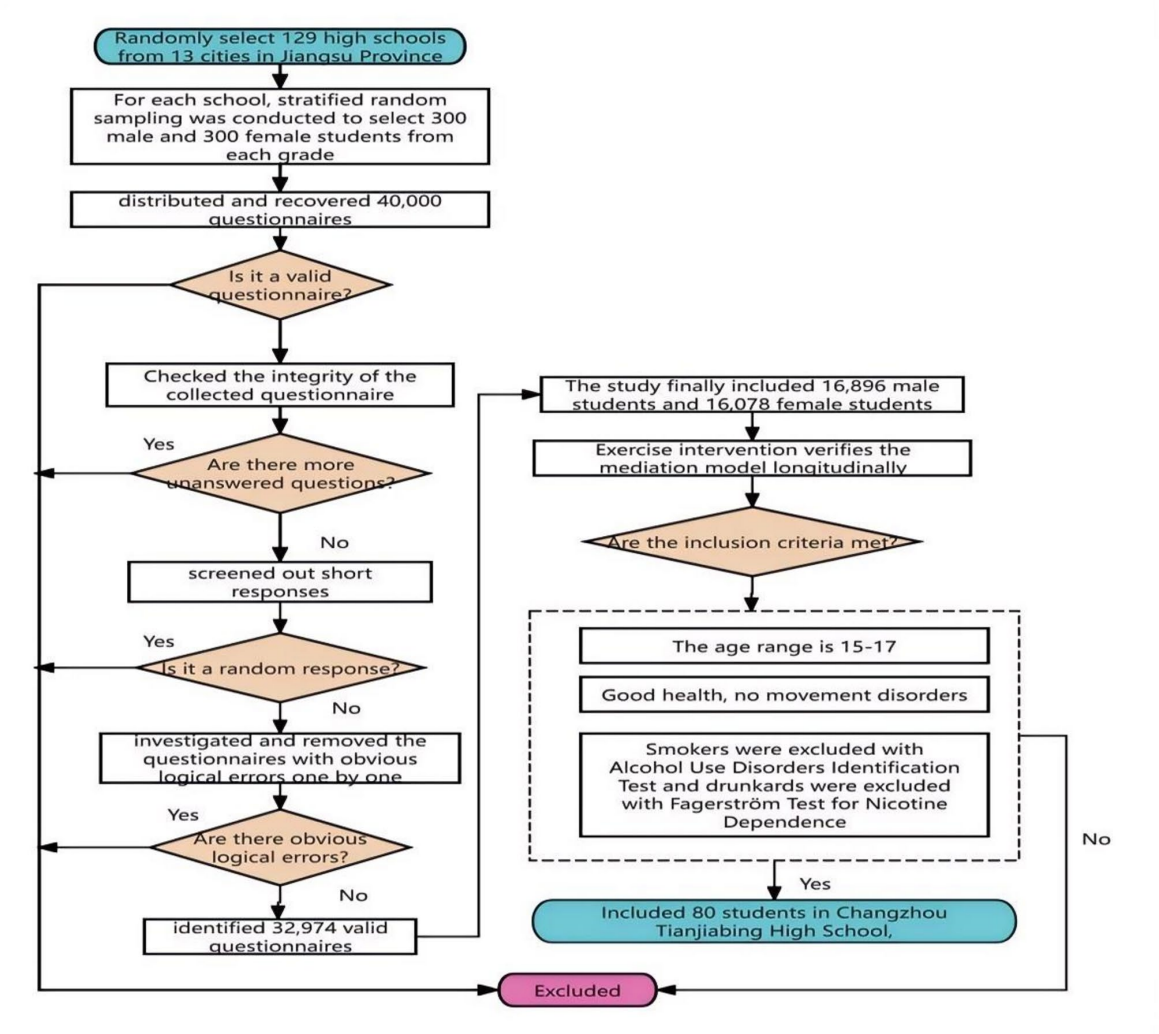
Flow chart of the study participant selection process

### Measurements

#### International physical activity questionnaire-short scale

The International Physical Activity Questionnaire Short scale (IPAQ) [32] comprises seven items designed to assess participants’ physical activity levels over the past week, investigating the frequency and duration of various intensity physical activities categorized into three domains: vigorous-intensity activities (e.g., running), moderate-intensity activities (e.g., brisk walking), and walking. Physical activity levels are quantified by calculating Metabolic Equivalent of Task (MET) values, where walking = 3.3 MET, moderate-intensity activities = 4.0 MET, and vigorous-intensity activities = 8.0 MET, using the formula: MET-min/week = activity intensity (MET) × duration (minutes) × frequency (days/week). These values are classified into three levels: low (failing to meet moderate or high activity criteria), moderate (engaging in vigorous-intensity activities for at least 3 days/week with ≥ 20 min/day, or moderate-intensity activities/walking for ≥ 5 days/week with ≥ 30 min/day, or any combination totaling ≥ 600 MET-minutes/week), and high (engaging in vigorous-intensity activities for ≥ 3 days/week with ≥ 1500 MET-minutes/week, or any combination of activities for ≥ 7 days/week totaling ≥ 3000 MET-minutes/week). During calculations, all durations are converted to minutes, excluding activities < 10 min, capping single sessions at 180 min, and limiting weekly durations to 1260 min per intensity level [33, 34]. The IPAQ has demonstrated good reliability and validity, with a Cronbach’s alpha coefficient of 0.92.

#### Pittsburgh sleep quality index scale

The Pittsburgh Sleep Quality Index Scale (PSQI) [35] was employed to assess participants’ sleep quality over the past month. Questionnaire includes seven “component” scores: subjective sleep quality, time to fall asleep, sleep duration, sleep efficiency, sleep disorders, hypnotic drugs, and daytime dysfunction. Each dimension is scored from 0 (no difficulty) to 3 (severe difficulty), with a global score ranging from 0 to 21. Higher scores indicate poorer sleep quality, categorized as excellent (PSQI < 3), moderate (PSQI 3–7), or poor (PSQI > 7). Specific scoring includes sleep latency (0: <15 min; 3: >60 min), sleep duration (0: >7 h; 3: <5 h), and sleep efficiency (0: >85%; 3: <65%). The PSQI demonstrates strong reliability, with a Cronbach’s alpha coefficient of 0.89.

#### Generalized anxiety disorder 7-item scale

The Generalized Anxiety Disorder 7-item Scale (GAD-7) [36] was utilized to assess participants’ anxiety symptoms over the past two weeks. The scale consists of 7 items, each rated on a four-point Likert scale ranging from 0 (not at all) to 3 (nearly every day), evaluating symptoms such as feeling anxious, being unable to control worrying, and experiencing irritability due to anxiety. The total score is calculated by summing the scores of all items, with the scale ranging from 0 to 21. Based on established clinical thresholds, the total score is categorized into five levels: no anxiety disorder (≤ 4), mild anxiety (5–9), moderate anxiety (10–14), and severe anxiety (15–21). The GAD-7 has demonstrated excellent reliability, with a Cronbach’s alpha coefficient of 0.88.

### Exercise intervention

We conducted an exercise intervention on Tianjiabing high school students and validated the questionnaire-based mediation model. This study employed a 2 (pre-test/post-test) × 2 (experimental/control) factorial design. Participants were allocated to matched cohorts through block randomization, with the experimental cohort comprising 40 students (22 males, 18 females) and the control cohort 40 students (19 males, 21 females). The 12-week structured exercise intervention protocol consisted of twice-weekly supervised sessions of 45-minute duration. Exercise intensity was regulated using Polar watch (M400, POLAR, USA) to maintain heart rate within 60–69% of age-adjusted maximum (maximum heart rate = 220 - age). To ensure the effect of intervention and the safety of subjects, sports professionals guided all interventions, and the same coach completed them. The experimental group received badminton combined with physical training, while the control group had only badminton intervention.

IPAQ, PSQI and GAD-7 were used to evaluate the physical activity level, sleep quality and anxiety level of high school students before and after exercise intervention.

### Statistical analysis

We conducted descriptive analysis to calculate the mean and standard deviation (SD) of physical activity, sleep quality, and anxiety. Independent t-tests is employed to explore differences in physical activity, sleep quality, and anxiety levels across various demographic characteristics. We set physical activity as the independent variable, sleep quality as the mediating variable, and anxiety as the dependent variable, and then developed regression analysis to determine the effect of physical activity and sleep quality on anxiety. SPSS macro PROCESS version 4.0 was used to analyze and test the mediating effect of sleep quality. All statistical analyses were conducted using Microsoft Excel 2019 and SPSS, Version 26.0 (SPSS Inc., Chicago, IL, USA).

Before the start of the study, we compared the baseline information of the experimental group and the control group using an independent sample t-test to ensure homogeneity. At the end of the study, we conducted repeated - measures analysis of variance to analyze the significance of the relevant variables before and after the intervention. Model 4 in SPSS macro PROCESS 4.0 ( *p* ≤ 0.05 was considered an indicator of statistical significance) was used again to construct and verify the mediation model in the cross-sectional study.

## Results

### Role of physical activity on anxiety among high school students: a cross-sectional study

#### Distribution of demographic characteristics in the sample

Table 1 lists the distribution of demographic characteristics in the sample.

**Table 1:** Descriptive statistics for all variables included in the current study (*n* = 32974)

Variable	Sample amount	Percent
**Gender**		
Male	16,896	51.2
Female	16,078	48.8
**Grade**		
10	10,974	33.3
11	9841	29.8
12	12,159	36.9
**Age**		
14–15	4051	12.3
16–17	24,783	75.2
18–20	4140	12.5
**BMI**		
< 17	1946	5.9
17 ≤ X < 24	24,724	74.0
24 ≤ X < 28	4418	13.4
≥ 28	1886	5.7
**Residential area**		
Urban	14,610	44.3
Rural	18,364	55.7
**Nationality**		
Han	32,362	98.1
Minority	612	1.9
**Only child**		
Yes	15,158	46.0
No	17,816	54.0

#### Descriptive analysis of physical activity, sleep quality and anxiety

**Table 2:** Physical activity, sleep quality and anxiety in general

Variable	Level	Sample amount(*n* = 32974)	Percentage
Physical activities	Low	12,003	36.40
	Medium	13,318	40.39
	High	7653	23.21
	Total	32,974	100.0
Sleep quality	Good	13,052	39.58%
	Medium	12,627	38.30%
	Poor	7295	22.12%
	Total	32,974	100.0
Anxiety	No anxiety(0–4)	13,650	41.40%
	Mild anxiety(5–9)	12,846	38.96%
	Moderate anxiety(10–13)	3779	11.46%
	Medium and severity anxiety (14–18)	2028	6.15%
	Severe anxiety(19–21)	671	2.03%
	Total	32,974	100.0

As shown in Tables 2, 12,003 (36.40%) students were in low physical activity level, 13,318 (40.39%) students were in medium physical activity level, and 7,653 (23.21%) students were in high physical activity level. Number of students with good, fair and poor sleep quality was 13,052 (39.58%), 12,627 (38.30%), and 7,295 (22.12%), respectively. When taking PSQI greater than 7 points as the critical value, the detection rate of sleep quality was 22.12%. There were 13,650 (41.40%) students without anxiety symptoms, 12,846 (38.96%) reported mild anxiety, 3,779 (11.46%) reported moderate anxiety, 2,028 (6.15%) reported moderate and severe anxiety, and 671 (2.03%) reported severe anxiety.

#### Demographic differences in physical activity, sleep quality and anxiety

**Table 3:** The differences of physical activity, sleep quality and anxiety in different demographic variables

variable	Physical activities (M ± SD)	t	*p*	Sleep quality (M ± SD)	t	*p*	Anxiety (M ± SD)	t	*p*
Male	1.95 ± 0.78	21.32	< 0.001	5.38 ± 2.90	10.47	< 0.001	5.65 ± 4.76	12.74	< 0.001
Female	1.78 ± 0.73			5.72 ± 2.90			6.32 ± 4.75		
Grade 10	1.93 ± 0.77	7.33	< 0.001	5.53 ± 2.89	0.77	0.555	5.93 ± 4.81	1.27	0.202
Grade 11	1.84 ± 0.76			5.57 ± 2.95			6.04 ± 4.87		
Grade 12	1.83 ± 0.75			5.55 ± 2.88			5.97 ± 4.65		
Urban	1.86 ± 0.76	0.90	0.369	5.54 ± 2.90	0.29	0.770	6.06 ± 4.80	2.67	0.008
Rural area	1.87 ± 0.76			5.55 ± 2.91			5.91 ± 4.75		
Only child	1.89 ± 0.75	4.47	< 0.001	5.37 ± 2.86	10.35	< 0.001	5.62 ± 4.78	12.45	< 0.001
Not only child	1.85 ± 0.77			5.70 ± 2.93			6.28 ± 4.74		

As shown in Table 3, No significant difference was found in physical activity level between regions (*t* = 0.90, *p* = 0.369). Males (*t* = 21.32, *p* < 0.001), Grade 10 students (*t* = 7.33, *p* < 0.001), and the only child (*t* = 4.47, *p* < 0.001) had significantly higher physical activity level. There were no difference in sleep quality among grades (*t* = 0.77, *p* = 0.555) and regions (*t* = 0.29, *p* = 0.770). Males (*t* = 10.47, *p* < 0.001) and the only child (*t* = 10.35, *p* < 0.001) had significantly better sleep quality. Students in different grades had similar anxiety level (*t* = 1.27, *p* = 0.202). Females (*t* = 12.74, *p* < 0.001), students in urban areas (*t* = 2.67, *p* < 0.001), and the non-only child (*t* = 12.45, *p* < 0.001) had significantly higher anxiety level.

#### Construction of mediating model of physical activity affecting anxiety

Table 4 lists the construction of mediating model of physical activity affecting anxiety.

**Table 4:** Analysis of mediating effect

Regression equation		Overall fitting index				
Predictive variable	Result variable	*R*	*R* ^2^	SE	t	β
Physical activities	Anxiety	0.036	0.001	0.035	-6.49	-0.036
Physical activities	Sleep quality	0.041	0.002	0.021	-7.54	-0.158
Physical activities	Anxiety	0.047	0.221	0.030	-3.34	-0.101
Sleep quality				0.008	96.58	0.771

On the premise of meeting the conditions of mediation analysis, physical activity was set as independent variable, sleep quality score as mediating variable and anxiety as dependent variable. Model 4 in SPSS macro PROCESS 4.0 was then performed and the results showed that physical activity significantly negatively predicted anxiety (*β*=-0.036, *t*=-6.49, *p* < 0.001) and sleep quality score (*β*=-0.158, *t*=-7.54, *p* < 0.001). After adding PSQI score to the mediation model, physical activity still negatively predicted anxiety (*β*=-0.101, *t*=-3.34, *p* < 0.001), while sleep quality score positively predicted anxiety (*β* = 0.771, *t* = 96.58, *p* < 0.001).

#### Verification of mediating model of physical activity affecting anxiety

**Table 5:** The mediation effect was tested by bootstrap method

	Effect	95%CI	Effect value
Overall effect	-0.224	[-0.292, -0.156]	100%
Direct effect	-0.101	[-0.162, -0.042]	45.42%
In-direct effect	-0.122	[-0.153, -0.090]	54.53%

**Effect** usually uses to measure the strength of the association between variables or the size of the effect of the intervention

**95%CI** = 95% confidence interval, these intervals will contain approximately 95% of the true population parameters

**Fig. 2:**
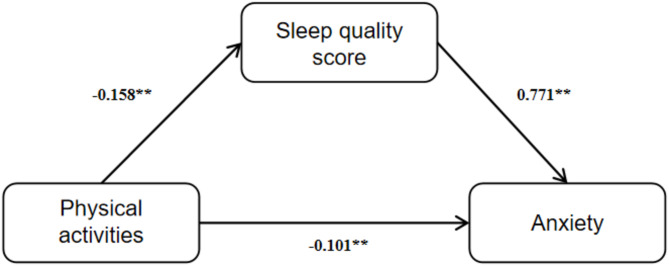
Path diagram of regression coefficient of mediation effect

Structural equation model (SEM) was developed to evaluate relationship between variables and Bootstrap method was used to test the mediating effect. As shown in Table 5; Fig. 2, physical activity had a significant direct effect on anxiety (*β*=-0.101, accounting for 45.42% of the total effect value). In addition, sleep quality score had a significant mediating effect between physical activity and anxiety (*β*=-0.122, accounting for 54.53% of the total effect value), which verified the hypothesis H4. Bootstrap was used to construct the confidence interval (*CI*) of the mediating effect, and the 95% *CI* was estimated. The results indicated that the direct effect of physical activity on anxiety, the indirect effect mediated by sleep quality and the total effect of the three were significant, with 95% *CIs* not including 0. Therefore, the direct and indirect effects were significant, proving the mediation effect of sleep quality.

### The effect of physical activity on anxiety through sleep quality among high school students: a longitudinal study

#### Effects of exercise intervention on physical activity, sleep quality and anxiety

##### Baseline comparison between experimental group and control group

**Table 6:** Differences of physical activity, sleep quality scores and anxiety in different groups of high school students before the study

Variable	EG(*n* = 40)	CG(*n* = 40)	t	*p*
Physical activities	1.93 ± 0.76	1.98 ± 0.73	-0.299	0.766
Sleep quality score	5.45 ± 3.77	5.42 ± 2.78	0.034	0.973
Anxiety	5.35 ± 4.38	4.97 ± 4.64	0.372	0.711

As shown in Table 6, there were no significant differences before intervention in physical activity (*p* = 0.766), PSQI score (*p* = 0.973) and anxiety (*p* = 0.711) between experimental group and control group, indicating the homogeneity of the two groups.

##### Effects of exercise intervention on physical activity, sleep quality and anxiety

**Fig. 3:**
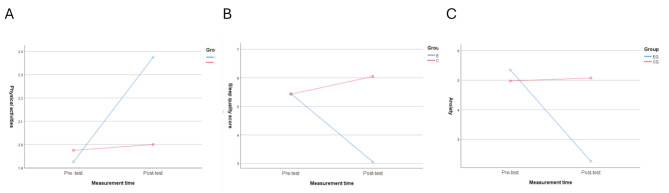
Changes of physical activity level, sleep quality scores and anxiety scores of high school students before and after exercise

Simple effect analysis in Fig. 3 took the time factor (pretest and posttest) and the group factor (experimental group and control group) as the independent variables, and the physical activity level, PSQI score, and GAD-7 score as the dependent variable. The Fig. 3A. showed that there was a main effect of time factor [*F*_(1,39)_ = 22.03, *p* < 0.05, η^2^ = 0.361], but no main effect of group factor [*F*_(1,39)_ = 1.00, *p* = 0.323, η^2^ = 0.025], and there was interaction between the two factors [*F*_(1,39)_ = 20.46, *p* < 0.05, η^2^ = 0.344]. The Fig. 3B. showed that there was main effect of both time factor [*F*_(1,39)_ = 8.61, *p* < 0.05, η^2^ = 0.181] and group factor [*F*_(1,39)_ = 7.74, *p* < 0.05, η^2^ = 0.166], and there was interaction between the two factors [*F*_(1,39)_ = 42.62, *p* < 0.05, η^2^ = 0.522]. The Fig. 3C. showed that there was a main effect of time factor [*F*_(1,39)_ = 23.88, *p* < 0.05, η^2^ = 0.380], but no main effect of group factor[*F*_(1,39)_ = 2.36, *p* > 0.05, η^2^ = 0.057], and there was interaction between the two factors [*F*_(1,39)_ = 24.56, *p* < 0.05, η^2^ = 0.386].

#### Construction and verification of the mediation model of physical activity on anxiety

##### Construction of mediating model of physical activity affecting anxiety

**Table 7:** Analysis of mediating effect

Regression equation		Overall fitting index				
Predictive variable	Result variable	*R*	*R* ^2^	SE	t	β
Physical activities	Anxiety	0.521	0.271	0.534	-5.39**	-2.879
Physical activities	Sleep quality	0.625	0.391	0.417	-7.09**	-2.956
Physical activities	Anxiety	0.632	0.400	0.625	-2.06*	-1.287
Sleep quality				0.132	4.07**	0.538

Longitudinal study data were then fitted by mediation model in study 1. On the basis of correlation analysis, physical activity was set as independent variable, sleep quality score as mediating variable and anxiety as dependent variable. Model 4 in SPSS macro PROCESS 4.0 was then performed and the results showed that physical activity significantly negatively predicted anxiety (*β*=-2.879, *t*=-5.39, *p* < 0.001) and sleep quality score (*β*=-2.956, *t*=-7.09, *p* < 0.001). After adding PSQI score to the mediation model, physical activity still negatively predicted anxiety (*β*=-1.287, *t*=-2.06, *p* < 0.05), while sleep quality score positively predicted anxiety (*β* = 0.538, *t* = 4.07, *p* < 0.001). See Table 7 for details.

##### Mediating effect of physical activity affecting anxiety

**Table 8:** The mediation effect was tested by bootstrap method

	Effect	95%CI	Effect value
Overall effect	-2.879	[-3.943, -1.816]	100%
Direct effect	-1.287	[-2.532, -0.042]	44.70%
In-direct effect	-1.592	[-2.486, -0.718]	55.29%

**Fig. 4:**
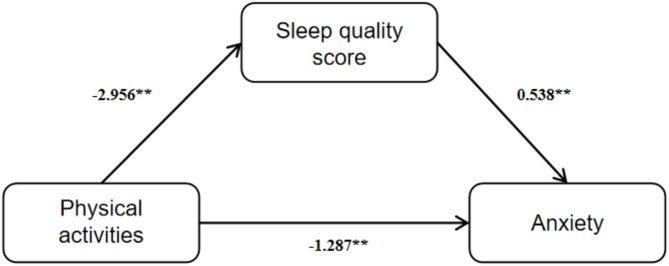
Path diagram of regression coefficient of mediation effect

Bootstrap method was used to test the mediating effect. As shown in Table 8; Fig. 4, physical activity had a significant direct effect on anxiety (*β*=-1.287, accounting for 44.70% of the total effect value). In addition, sleep quality score had a significant mediating effect between physical activity and anxiety (*β*=-1.287, accounting for 55.29% of the total effect value). Bootstrap was used to construct the *CI* of the mediating effect, and the 95% *CI* was estimated. The results indicated that the direct effect of physical activity on anxiety, the indirect effect mediated by sleep quality and the total effect of the three were significant, with 95% *CI*s not including 0. Therefore, the direct and indirect effects were significant, proving the mediation effect of sleep quality.

## Discussion

### Relationship between physical activity, sleep quality and anxiety among high school students

#### Physical activity positively predicts sleep quality

Chronic sedentary behavior and exercise deficiency in high school students contribute to diminished physical activity levels, heightening susceptibility to sleep disorders and anxiety-related pathologies [37]. Convergent evidence from collegiate populations demonstrates a robust positive association between physical activity engagement and sleep quality metrics (*r* = 0.856) [38]. Structured aerobic interventions yield differential therapeutic effects, with 10-week jogging protocols producing significant improvements in sleep architecture—particularly among adolescents exhibiting compromised sleep efficiency and prolonged sleep latency [39]. Most current studies have focused on college students rather than high school students. In both cross-sectional study and longitudinal study, we observed that there was a significant negative correlation between physical activity level and sleep quality score, that is, the higher the physical activity level, the better the sleep quality. The above results validated our hypothesis H2 and were consistent with Hosker’s study [40]. High level of physical activity could significantly enhance the satisfaction of basic psychological needs of high school students, thus positively predicting sleep quality. Elevated physical activity levels modulate neuroendocrine pathways through β-endorphin upregulation and enhanced endogenous opioid peptide synthesis, thereby optimizing metabolic homeostasis and affective states—mechanisms that ultimately attenuate sleep-disruptive psychological factors and improve sleep architecture [41, 42]. Neuroimaging evidence further identifies structural and functional aberrations in poor sleepers, characterized by increased regional homogeneity (ReHo) in the dorsolateral superior frontal gyrus and left medial/middle frontal regions, alongside elevated amplitude of low-frequency fluctuation (ALFF) within the right orbitofrontal cortex, cingulo-paracingulate complex, insular cortex, and dorsolateral prefrontal circuitry [43]. Conversely, sustained physical activity engagement promotes neuroplastic adaptations in sleep-disordered individuals, demonstrating restorative effects on corticolimbic network integrity [44, 45].

#### Relationship between physical activity and anxiety

Physical activity constitutes a neurobehavioral intervention for anxiety amelioration in adolescents [46], as empirically validated through multisite trials by Shepherd et al. [47] and Wen et al. [48]. Longitudinal investigations demonstrate sustained engagement in physical activity confers longitudinal anxiety reduction across the adolescent developmental trajectory [49]. Meta-analytic evidence further establishes that structured aerobic protocols (20-minute sessions, 2–3 weekly, ≥ 4 weeks) enhance both physical activity metrics and anxiety symptom reduction through dose-response relationships [50]. Our findings corroborate this inverse association between physical activity engagement and anxiety severity—demonstrating an inverse dose-response relationship consistent with extant literature and confirming hypothesis H3. Mechanistically, this effect may arise from physical activity-induced HPA axis downregulation, which modulates cortisol dynamics to enhance neural oscillations (ReHo/ALFF) in the hippocampal dentate gyrus [51]. Furthermore, physical activity mediates anxiety reduction through dual neurobiological pathways: affective regulation via attentional bias modification and monoaminergic-endogenous opioid system neuromodulation [52, 53].

#### Relationship between sleep quality and anxiety

Within the positive psychology framework, sleep quality emerges as a modifiable protective factor demonstrating robust inverse predictive validity for anxiety severity. Empirical investigations into academic stress-anxiety dynamics reveal sleep quality’s mediating role, where elevated Pittsburgh Sleep Quality Index (PSQI) scores (indicative of poorer sleep) positively correlate with anxiety symptom escalation [54, 55]. This relationship manifests bidirectionally, as psychometric assessments confirm significant positive associations between PSQI scores and anxiety sensitivity indices in adolescents, while heightened anxiety states reciprocally exacerbate sleep architecture disturbances [56, 57]. In this study, both study 1 and study 2 found that there was a significant positive correlation between PSQI scores and GAD-7 scores, that is, the higher the sleep quality, the lower the anxiety level of high school students, which verified our hypothesis H1. The observed effects may be mediated through interconnected neuropsychosocial pathways, where sleep quality exerts anxiolytic effects via synergistic interactions among social support systems, emotion regulation competencies, and psychological resilience mechanisms [58, 59]. At the neurocircuitry level, sleep quality-associated ventromedial prefrontal cortex (vmPFC) hyperactivation exerts inhibitory control over amygdalohippocampal reactivity, simultaneously attenuating fear memory encoding while potentiating extinction learning processes. Concurrently, augmented functional connectivity within limbic networks facilitates cortically mediated emotional regulation [60]. On the molecular level, preserved sleep integrity prevents astrocytic dystrophy through bidirectional modulation of hippocampal astrocyte phenotypes—suppressing pro-inflammatory A1 astrocyte proliferation while enhancing neuroprotective A2 astrocyte activity—thereby reducing apoptotic neuronal loss and associated anxiety pathogenesis [61].

### The mediating role of sleep quality in physical activity improving anxiety among high school students

This study confirmed the mediating role of sleep quality in the relationship between physical activity and anxiety in high school students, which verified our hypothesis H4. The main way of physical activity to prevent and relieve anxiety was to exert its physiological, psychological and sociological effects, that is, promote the secretion of neurotransmitters such as endorphins and improve mood state and psychological resilience to improve sleep quality [62, 63]. A growing body of evidence on the causes or paths of physical activity improving anxiety is from a theoretical point of view, but very few have focused on high school students. Study 1 delineates dual pathways through which physical activity alleviates adolescent anxiety: direct anxiolytic effects and sleep quality-mediated indirect effects. Study 2’s randomized controlled trial revealed significant time × group interaction effects following the 12-week intervention, manifesting as increased International Physical Activity Questionnaire (IPAQ) scores concomitant with reduced Pittsburgh Sleep Quality Index (PSQI) and Generalized Anxiety Disorder-7 (GAD-7) metrics. These convergent findings validate hypothesis H4, demonstrating that structured exercise interventions enhance physical activity levels to improve both sleep architecture and anxiety symptomatology—a pattern congruent with established behavioral neuroscience frameworks [64, 65]. Adolescents with elevated physical activity engagement demonstrated superior sleep maintenance (reduced nocturnal awakenings), extended total sleep time, and accelerated sleep onset latency [66]. This phenomenon may be attributed to physical activity’s multifactorial mechanisms: academic stress buffering, psychophysiological relaxation, fatigue attenuation, self-efficacy enhancement, digital addiction prophylaxis, and circadian entrainment via melatonin regulation [67].

Optimal sleep quality demonstrates neuroprotective properties through prospective negative prediction of adolescent anxiety and affective dysregulation. Physiological psychology research identifies sleep deprivation as a potentiator of anxiety perception via disrupted hypothalamic-pituitary-adrenal axis modulation [68], while positive psychology frameworks reveal sleep-mediated anxiety reduction through tetrad mechanisms: enhanced positive affect, strengthened resilience traits, enriched environmental adaptability, and optimized social support networks [69]. Mechanistically, sleep quality modulates corticolimbic circuitry via circadian-aligned cortisol rhythmicity that stabilizes emotional reactivity thresholds, while augmented prefrontal-limbic functional connectivity coupled with attenuated amygdala activation synergistically facilitates anxiety mitigation [70]. Neuroimaging evidence further corroborates that sleep optimization attenuates trait anxiety’s impact on emotional processing through ventromedial prefrontal cortex-mediated suppression of limbic hyperreactivity [71].

### Limitations

This investigation acknowledges three principal limitations requiring scholarly attention. The Pittsburgh Sleep Quality Index’s multidimensional construct necessitates granular mediation analyses to elucidate individual subcomponent contributions, as the current operationalization may obscure differential pathway effects. Secondly, the geographically restricted sample constrains ecological validity, necessitating replication across demographically diverse cohorts to assess cross-population generalizability. Finally, the mediation framework excludes critical psychosocial factors, behavioral dependencies, and neuroendocrine pathways that may interactively modulate the physical activity-anxiety nexus. Subsequent research should employ multivariate mediation networks incorporating machine learning approaches to disentangle these polygenic-environmental interactions. In future research, we will employ multivariate regression analysis or structural equation modeling to control for multiple confounding factors, while also utilizing stratified analysis to conduct a comprehensive exploration from various perspectives. Fourth, there is a reliance on self-reported measurement methods and a lack of objective data. This study mainly used subjective self-reported questionnaires to assess sleep quality and anxiety conditions, which may introduce recall bias or social desirability bias. In addition, although the study proposed physiological mechanisms to explain these relationships, these hypotheses have not been directly verified through objective biomarkers. Future research should integrate multimodal data collection methods, combining subjective reports with wearable devices, biochemical tests, or neuroimaging techniques, in order to strengthen causal inference and mechanism verification.

### Suggestions for future research

1. Researchers can develop a multimodal data integration system and create a dynamic monitoring platform that integrates wearable devices and AI emotion recognition technology. This platform can track in real time the dynamic relationships among the physical activity intensity, sleep cycles, and anxiety states of high school students, and construct a three-dimensional data model. By using machine learning algorithms to analyze the nonlinear relationships among the data, and identifying the changes in the intensity of the mediating pathway of " physical activity → sleep quality → anxiety” at different time points through time series models, a basis can be provided for dynamic intervention.
2. Researchers can design personalized intervention programs. Generate personalized exercise plans based on the AI recommendation system: Dynamically adjust the type, time, and frequency of exercise according to students’ physical fitness levels, daily routines, and baseline anxiety values. Design immersive exercise scenarios in combination with virtual reality technology, enhance exercise compliance through environmental simulation, and at the same time, optimize the sleep induction program by using biofeedback technology.
3. In future research, the following measures can be taken to reduce the influence of other factors on the relationship among the three aspects. For example, conduct multi-dimensional data collection by simultaneously measuring variables such as family economic status, personality traits, academic pressure, and the use of digital devices, and establish a comprehensive database. Use objective monitoring devices (such as fitness trackers) to record the amount of physical exercise and sleep quality, so as to reduce self-reporting biases. Adopt longitudinal and intervention studies, carry out long-term follow-up research, and distinguish the causal direction. For instance, determine whether poor sleep leads to a decrease in physical exercise or insufficient physical exercise causes sleep problems.

## Conclusions

1. The enhancement of physical activity level could significantly improve sleep quality and relieve anxiety among high school students.
2. Sleep quality could negatively predict anxiety.
3. Sleep quality played a significant mediating role in the relationship between physical activity and anxiety among high school students.

## Electronic supplementary material

Below is the link to the electronic supplementary material.

Supplementary Material 1

## Data Availability

The datasets can be made available to any interested person(s) contacting the corresponding author via email.

## References

[1] Collins DP, Jagim AR, Sowders JP, et al. Athletic disruptions caused by the COVID-19 pandemic negatively affect high school student-athletes social-emotional well-being. Med (Baltim). 2022;101(51):e31890. 10.1097/MD.0000000000031890 PMC9794343 36595767

[2] Quek TT, Tam WW, et al. The global prevalence of anxiety among medical students: a meta-analysis. Int J Environ Res Public Health. 2019;16(15):2735. 31370266 10.3390/ijerph16152735 PMC6696211

[3] Jiang B, He J. Study of sleep quality and correlative factors in 344 students of key senior middle school. Chin J Prev Control Chronic Dis. 2008;16(8):384–7.

[4] Li XW. A comparative study on related factors of sleep quality, anxiety and depression between urban and rural senior high school students. Master thesis. Shandong University. 2009.

[5] Hong X, Liang YQ, Wang ZY, et al. Association of sleep status with depression among high school students in Nanjing. Chin J Public Health. 2007;23(11):1322–4.

[6] Cao XJ, Zhang QY, Liu XQ. Cross-lagged relationship between physical activity time, openness and depression symptoms among adolescents: evidence from China. Int J Mental Health Promotion. 2023;25:1009–18.

[7] Liu X, Wang J. Depression, anxiety, and student satisfaction with university life among college students: a cross-lagged study. Humanit Social Sci Commun. 2024;11:1172.

[8] Musliner KL, Munk-Olsen T, Eaton WW, et al. Heterogeneity in long-term trajectories of depressive symptoms: patterns, predictors and outcomes. J Affect Disord. 2016;192:199–211. 26745437 10.1016/j.jad.2015.12.030 PMC4761648

[9] Nandi A, Beard JR, Galea S. Epidemiologic heterogeneity of common mood and anxiety disorders over the lifecourse in the general population: a systematic review. BMC Psychiatry. 2009;9(1):31. 19486530 10.1186/1471-244X-9-31 PMC2700109

[10] Kessler RC, Merikangas KR, Wang PS. Prevalence, comorbidity, and service utilization for mood disorders in the United States at the beginning of the twenty-first century. Ann Rev Clin Psychol. 2010;6:137–58. 10.1146/annurev.clinpsy.3.022806.091444 17716051

[11] Bruni O, Ferini-Strambi L, Giacomoni E, et al. Herbal remedies and their possible effect on the GABAergic system and sleep. Nutrients. 2021;13(2):530. 33561990 10.3390/nu13020530 PMC7914492

[12] Richards A, Kanady JC, Neylan TC. Sleep disturbance in PTSD and other anxiety-related disorders: an updated review of clinical features, physiological characteristics, and psychological and neurobiological mechanisms. Neuropsychopharmacol. 2020;45(1):55–73. 10.1038/s41386-019-0486-5 PMC6879567 31443103

[13] Xiao Q, Zhou X, Wei P, et al. A new GABAergic somatostatin projection from the BNST onto accumbal parvalbumin neurons controls anxiety. Mol Psychiatry. 2021;26(9):4719–41. 32555286 10.1038/s41380-020-0816-3 PMC8589681

[14] Puteikis K, Mameniskýtė A, Mamenišienė R, Sleep, Quality. Mental health and learning among high school students after reopening schools during the COVID-19 pandemic: results of a cross-sectional online survey. Int J Environ Res Public Health. 2022;19(5):2553. 35270245 10.3390/ijerph19052553 PMC8909739

[15] Yu YB. A study on the correlation between physical activity, sleep quality and life satisfaction of senior high school students – a case study of Xuchang city. Master thesis. Shanghai University of Sport. 2021.

[16] Hasson R, Sallis JF, Coleman N, et al. COVID-19: implications for physical activity, health disparities, and health equity. Am J Lifestyle Med. 2021;16(4):420–33. 35855783 10.1177/15598276211029222 PMC9283961

[17] Parker H, Burkart S, Reesor-Oyer L, et al. Feasibility of measuring screen time, activity, and context among families with preschoolers: intensive longitudinal pilot study. JMIR Formative Res. 2022;6(9):e40572. 10.2196/40572 PMC9562053 36173677

[18] Lu XP, Tan CQ, Tan YQ. Relationship between physical activity, sleep and anxiety among junior high school students in Yangzhou. Chin J School Health. 2022;43(8):1185–8.

[19] Fa JJ. Physical activity, screen time and sleep duration: combined associations with depression in college students. J Nanjing Normal Univ (Natural Sci Edition). 2021;44(4):135–9.

[20] Cui GH, Li SJ, Yin YT, et al. Effects of sedentary behavior and sleep quality on cognitive function among elderly people. Mod Prev Med. 2020;47(18):3339–42.

[21] Shen Q, Wang S, Liu Y, et al. The chain mediating effect of psychological inflexibility and stress between physical exercise and adolescent insomnia. Sci Rep. 2024;14(1):24348. 39420219 10.1038/s41598-024-75919-8 PMC11486977

[22] Xiao T, Pan M, Xiao X, et al. The relationship between physical activity and sleep disorders in adolescents: a chain-mediated model of anxiety and mobile phone dependence. BMC Psychol. 2024;12(1):751. 39695835 10.1186/s40359-024-02237-z PMC11658458

[23] Zhang YF. A study on the relationship between health fitness, social physical activity and sedentary behavior in senior high school students. Master thesis. East China Normal University. 2022.

[24] Schmits E, Dekeyser S, Klein O, et al. Psychological distress among students in higher education: one year after the beginning of the COVID-19 pandemic. Int J Environ Res Public Health. 2021;18(14):7445. 34299896 10.3390/ijerph18147445 PMC8308017

[25] Villani L, Pastorino R, Molinari E, et al. Impact of the COVID-19 pandemic on psychological well-being of students in an Italian University: a web-based cross-sectional survey. Global Health. 2021;17(1):39. 33823897 10.1186/s12992-021-00680-w PMC8022300

[26] Gao L. The compilation and preliminary application of group guidance manual for physical activity combined with test anxiety. Master thesis. Yunnan Normal University. 2016.

[27] Liu Y, Xiao T, Zhang W, et al. The relationship between physical activity and internet addiction among adolescents in Western China: a chain mediating model of anxiety and inhibitory control. Psychol Health Med. 2024;29(9):1602–18. 38770920 10.1080/13548506.2024.2357694

[28] Liu Y, Jin Y, Chen J, et al. Anxiety, inhibitory control, physical activity, and internet addiction in Chinese adolescents: a moderated mediation model. BMC Pediatr. 2024;24(1):663. 39407215 10.1186/s12887-024-05139-6 PMC11481747

[29] Kawachi I, Berkman LF. Social ties and mental health. J Urban Health. 2001;78(3):458–67. 11564849 10.1093/jurban/78.3.458 PMC3455910

[30] Ji C, Yang J, Lin L, et al. Anxiety and sleep quality amelioration in college students: a comparative study between team sports and individual sports. Behav Sci (Basel). 2022;12(5):149. 35621446 10.3390/bs12050149 PMC9138125

[31] Kredlow MA, Capozzoli MC, Hearon BA, et al. The effects of physical activity on sleep: a meta-analytic review. J Behav Med. 2015;38(3):427–49. 25596964 10.1007/s10865-015-9617-6

[32] Qu NN, Li KJ. Study on the reliability and validity of international physical activity questionnaire (Chinese vision, IPAQ). Chin J Epidemiol. 2004;25(3):265–8. 15200945

[33] An HY, Chen W, Wang CW, et al. The relationships between physical activity and life satisfaction and happiness among young, middle-aged, and older adults. Int J Environ Res Public Health. 2020;17(13):4817. 32635457 10.3390/ijerph17134817 PMC7369812

[34] Altunalan T, Arslan E, Ocakoglu AO. The relationship between physical activity level and timing and sleep quality and hygiene in healthy individuals: a cross-sectional study. BMC Public Health. 2024;24(1):3261. 39581962 10.1186/s12889-024-20708-1 PMC11587700

[35] Liu XC, Tang MQ, Hu L, et al. Reliability and validity of the Pittsburgh sleep quality index. Chin J Psychiatry. 1996;29(2):103–7.

[36] Spitzer RL, Kroenke K, Williams JB, et al. A brief measure for assessing generalized anxiety disorder: the GAD-7. Arch Intern Med. 2006;166(10):1092–7. 16717171 10.1001/archinte.166.10.1092

[37] Delgado-Floody P, Caamaño Navarrete F, Chirosa-Ríos L, et al. Exercise training program improves subjective sleep quality and physical fitness in severely obese bad sleepers. Int J Environ Res Public Health. 2022;19(21):13732. 36360611 10.3390/ijerph192113732 PMC9658425

[38] Li D, Li X. Independent and combined associations between physical activity and sedentary time with sleep quality among Chinese college students. Int J Environ Res Public Health. 2022;19(11):6697. 35682279 10.3390/ijerph19116697 PMC9179993

[39] Hedlund ER, Villard L, Lundell B, et al. Physical exercise may improve sleep quality in children and adolescents with Fontan circulation. Cardiol Young. 2019;29(7):922–9. 31218992 10.1017/S1047951119001136

[40] Hosker DK, Elkins RM, Potter MP. Promoting mental health and wellness in youth through physical activity, nutrition, and sleep. Child Adolesc Psychiatr Clin N Am. 2019;28(2):171–93. 30832951 10.1016/j.chc.2018.11.010

[41] Paluska SA, Schwenk TL. Physical activity and mental health: current concepts. Sports Med. 2000;29(3):167–80. 10739267 10.2165/00007256-200029030-00003

[42] Deslandes A, Moraes H, Ferreira C, et al. Exercise and mental health: many reasons to move. Neuropsychobiology. 2009;59(4):191–8. 19521110 10.1159/000223730

[43] Kang D, Qin Z, Wang W, et al. Brain functional changes in Tibetan with obstructive sleep apnea hypopnea syndrome: a resting state fMRI study. Med (Baltim). 2020;99(7):e18957. 10.1097/MD.0000000000018957 PMC7035052 32049791

[44] Zhao JL, Jiang WT, Wang X, et al. Exercise, brain plasticity, and depression. CNS Neurosci Ther. 2020;26(9):885–95. 32491278 10.1111/cns.13385 PMC7415205

[45] Zhang X, Zong B, Zhao W, et al. Effects of mind-body exercise on brain structure and function: a systematic review on MRI studies. Brain Sci. 2021;11(2):205. 33562412 10.3390/brainsci11020205 PMC7915202

[46] McGuine TA, Biese KM, Petrovska L, et al. Mental health, physical activity, and quality of life of US adolescent athletes during COVID-19-related school closures and sport cancellations: a study of 13,000 athletes. J Athl Train. 2021;56(1):11–9. 33290516 10.4085/1062-6050-0478.20 PMC7863599

[47] Shepherd HA, Evans T, Gupta S, et al. The impact of COVID-19 on high school student-athlete experiences with physical activity, mental health, and social connection. Int J Environ Res Public Health. 2021;18(7):3515. 33805249 10.3390/ijerph18073515 PMC8036482

[48] Wen X, Lin Y, Liu Y, et al. A latent profile analysis of anxiety among junior high school students in less developed rural regions of China. Int J Environ Res Public Health. 2020;17(11):4079. 32521646 10.3390/ijerph17114079 PMC7312008

[49] Dishman RK, McIver KL, Dowda M, Pate RR. Declining physical activity and motivation from middle school to high school. Med Sci Sports Exerc. 2018;50(6):1206–15. 29298219 10.1249/MSS.0000000000001542 PMC5953776

[50] Tang Z, Wang Y, Liu J, et al. Effects of aquatic exercise on mood and anxiety symptoms: a systematic review and meta-analysis. Front Psychiatry. 2022;13:1051551. 36465296 10.3389/fpsyt.2022.1051551 PMC9714032

[51] Voss MW, Vivar C, Kramer AF, et al. Bridging animal and human models of exercise-induced brain plasticity. Exploring exercise as an avenue for the treatment of anxiety disorders. Expert Rev Neurother. 2013;17(10):525–44. 10.1016/j.tics.2013.08.001 PMC4565723 24029446

[52] Neil-Sztramko SE, Caldwell H, Dobbins M. School-based physical activity programs for promoting physical activity and fitness in children and adolescents aged 6 to 18. Cochrane Database Syst Rev. 2021;9(9):CD007651. 34555181 10.1002/14651858.CD007651.pub3 PMC8459921

[53] DeBoer LB, Powers MB, Utschig AC, et al. Exploring exercise as an avenue for the treatment of anxiety disorders. Exploring exercise as an avenue for the treatment of anxiety disorders. Expert Rev Neurother. 2012;12(8):1011–22. 23002943 10.1586/ern.12.73 PMC3501262

[54] Gao HJ. Study on the relationship between academic stress, rumination, sleep quality and negative emotions in high-altitude high school students. Master thesis. Qinghai Normal University. 2022.

[55] Yang JW, Liang J, Liu JW, et al. Sleep quality and its correlation with anxiety and depressive symptoms in patients with anxiety disorder and depressive disorder. J Neurosci Mental Health. 2022;22(03):161–71.

[56] Zhou YH, Qin Y, Xie HQ. Correlation analysis of anxiety sensitivity and sleep status in high school students. Teach Adm. 2013;2013(36):91–3.

[57] Castelnuovo A, Mombelli S, Bottoni D, et al. Quality of sleep is the only predictor of suicide during Covid-19 lockdown in university students? Sleep. 2021;44(2):A272.

[58] Zhang PC, Li X, Han WY, et al. The effect of lack of sleep on negative emotion in primary and secondary school students: a chain mediation model. Psychol Dev Educ. 2023;29(3):402–9.

[59] Cai HJ, Guo JH, Lai YQ. Analysis on the relationship between sleep quality and anxiety symptoms of college students: the mediating role of resilience. J Fujian Med Univ (Social Sci Edition). 2022;23(5):24–9.

[60] Zhang J, Zhang HY, Li H, et al. The effect of sleep on fear learning and its cognitive neural mechanisms. Adv Psychol Sci. 2022;31(4):631–40.

[61] Hong H, Lu X, Lu Q, et al. Potential therapeutic effects and pharmacological evidence of sinomenine in central nervous system disorders. Front Pharmacol. 2022;13:1015035. 36188580 10.3389/fphar.2022.1015035 PMC9523510

[62] Bolijn S, Lucassen PJ. How the body talks to the brain; peripheral mediators of physical activity-induced proliferation in the adult hippocampus. Brain Plast. 2015;1(1):5–27. 29765833 10.3233/BPL-150020 PMC5939189

[63] Fleming KM, Coote SB, Herring MP. Home-based pilates for symptoms of anxiety, depression and fatigue among persons with multiple sclerosis: an 8-week randomized controlled trial. Multiple Scler J. 2021;27(14):2267–79. 10.1177/13524585211009216 PMC8597189 33870785

[64] Han SJ, Han X, Duan H, et al. Effects of exercise intervention on sleep and physical activity of obese adolescents. Wushu Stud. 2022;7(03):140–3.

[65] Gong MJ, Fu J, Hu XF. Meta-analysis on the effects of exercise intervention on sleep disorder. China Sport Sci Technol. 2020;56(03):22–31.

[66] Lang C, Brand S, Feldmeth AK, et al. Increased self-reported and objectively assessed physical activity predict sleep quality among adolescents. Physiol Behav. 2013;120:46–53. 23851332 10.1016/j.physbeh.2013.07.001

[67] Yang J, Lei YJ. Correlation between sleep quality and physical activity in high school students. Teach Adm. 2014;2014(09):53–5.

[68] Riemann D, Krone LB, Wulff K, et al. Sleep, insomnia, and depression. Neuropsychopharmacol. 2020;45(1):74–89. 10.1038/s41386-019-0411-y PMC6879516 31071719

[69] Grey I, Arora T, Thomas J, et al. The role of perceived social support on depression and sleep during the COVID-19 pandemic. Psychiatry Res. 2020;293:113452. 32977047 10.1016/j.psychres.2020.113452 PMC7500407

[70] Ren ZH, Zhao ZY, Yu XL, et al. Testosterone and aggressive behavior in juvenile offenders with antisocial tendency: the mediation effect of hostile attention bias and the moderation effect of cortisol. Acta Physiol Sinica. 2020;52(11):1288–300.

[71] Goldstein AN, Greer SM, Saletin JM, et al. Tired and apprehensive: anxiety amplifies the impact of sleep loss on aversive brain anticipation. J Neurosci. 2013;33(26):10607–15. 23804084 10.1523/JNEUROSCI.5578-12.2013 PMC3693050

