# Supplementary material for: The effect of physical activity on anxiety through sleep quality among Chinese high school students: evidence from cross-sectional study and longitudinal study
Source: BMC Psychiatry. 2025 May 16;25:495. doi: 10.1186/s12888-025-06909-x (PMC12084996; doi:10.1186/s12888-025-06909-x)
Supplement: Supplementary file 1 — Supplementary Material 1 [file 12888_2025_6909_MOESM1_ESM.pdf]

医学伦理学审查报告

我院拟开展“ 精准运动改善青少年抑郁症的模式构建及应用研究 ”科研项目的相关研究，院伦理委员会对该项目的相关医学伦理学问题进行了审查。

项目信息：

项目名称：精准运动改善青少年抑郁症的模式构建及应用研究（国家社会科学基金教育学青年课题）

项目编号：CLA200279

承担单位：扬州大学体育学院

项目负责人：陈祥和　　　　　　职称：副教授

研究起止日期：2020 年 9 月--------2023 年 12 月

伦理编号：YZUHL2020018

审查评议意见：

根据中华人民共和国食品药品监督管理局颁布实施的《药物临床试验管理规定》以及《赫尔辛基宣言》和国际医学科学组织委员会颁布的《人体生物医学研究国际伦理指南》的道德原则，即公正、尊重人格、力求使受试者最大程度受益和尽可能避免伤害，经本伦理委员会讨论，认为该项目研究符合上述原则，准予通过。

结论：

同意该项研究的工作按计划进行。

扬州大学护理学院伦理委员会

2020 年 4 月 1 日

Medical ethics review report

Our school plans to carry out the scientific research project named

" Research on Model Construction and Application of Precise Exercise to Improve Adolescent Depression ", and the ethics committee of our school has reviewed the relevant medical ethics issues of the project.

Project information:

Project Name: Research on Model Construction and Application of Precise Exercise to Improve Adolescent Depression（National Social Science Foundation Educational Youth Project）

Project Number: CLA200279

Responsible Unit: Yangzhou University Institute of Physical Education

Project Leader: Chen Xianghe Title: Associate Professor

Start and end date of the study: YZUHL2020018

Review comments:

According to the ethical principles of "Regulations on the Administration of Clinical Trials of Drugs"issued by Food and Drug Administration of the People's Republic of China, and "Declaration of Helsinki" and "International Ethics Guide for Biomedical Research of Human Body"issued by the Committee of the International Medical Science Organization. Namely, justice, respect for personality, striving to maximize the benefit of the subjects and avoiding harm as much as possible. Our ethics committee discussed the project study was in line with the above principles then was approved.

Conclusion:

It was agreed that the work of the study can be carried out as planned.

Ethics Committee of School of Nursing, Yangzhou University

Date: April 1, 2020

扬州大学护理学院伦理委员会
